# Basis and applicability of noninvasive inverse electrocardiography: a comparison between cardiac source models

**DOI:** 10.3389/fphys.2023.1295103

**Published:** 2023-12-13

**Authors:** Jeanne van der Waal, Veronique Meijborg, Ruben Coronel, Rémi Dubois, Thom Oostendorp

**Affiliations:** ^1^ Department of Clinical and Experimental Cardiology, Amsterdam University Medical Centers, Amsterdam, Netherlands; ^2^ IHU Liryc, Electrophysiology and Heart Modeling Institute, Fondation Bordeaux Université, Pessac, France; ^3^ Donders Institute for Brain, Cognition and Behaviour, Radboud University Medical Centre, Nijmegen, Netherlands

**Keywords:** noninvasive mapping, electrocardiography, cardiac source models, electrocardiographic imaging, inverse electrocardiography, ECGI

## Abstract

The body surface electrocardiogram (ECG) is a direct result of electrical activity generated by the myocardium. Using the body surface ECGs to reconstruct cardiac electrical activity is called the inverse problem of electrocardiography. The method to solve the inverse problem depends on the chosen cardiac source model to describe cardiac electrical activity. In this paper, we describe the theoretical basis of two inverse methods based on the most commonly used cardiac source models: the epicardial potential model and the equivalent dipole layer model. We discuss similarities and differences in applicability, strengths and weaknesses and sketch a road towards improved inverse solutions by targeted use, sequential application or a combination of the two methods.

## 1 Introduction

Cardiac arrhythmias are the result of the combined presence of a structural and/or functional pre-existing condition (the arrhythmogenic substrate) and an initiating factor (the trigger) (Coumel, 1987). The 12-lead electrocardiogram (ECG) provides a relatively quick and easy way to establish major conditions such as myocardial ischemia or electrolyte imbalance. However, the resolution of the standard ECG is too low to detect detailed information of the electrical activity at the myocardial level. Local information of the arrhythmogenic substrate is important to target therapy for prevention of life-threatening cardiac arrhythmias (Bakker et al., 1988; Stevenson and Soejima, 2007; Shivkumar, 2019). Information of the arrhythmogenic substrate is often gained by imaging techniques (MRI, CT) or invasive endo- or epicardial mapping procedures. The latter yields the most detailed information (Shivkumar, 2019), but is time consuming, costly and poses a burden to the patient (Stevenson et al., 1998; Santangeli and Marchlinski, 2016). In addition, clinically relevant arrhythmia often cannot be studied, because it is not present during the medical exam or is not stable enough to be mapped (Santangeli and Marchlinski, 2016).

A method to overcome the limitations of the standard ECG, as well as those of invasive mapping, is Electrocardiographic Imaging (ECGI). This is a noninvasive mapping technique, that allows a bedside diagnosis of arrhythmogenic substrates from the body surface ECG recorded in many leads (Cuppen et al., 1984; Rudy, 1999). It allows simultaneous mapping of the relevant parameters in the wake patient, potentially also during polymorphic, hemodynamically ill-tolerated, and sustained arrhythmias (Pereira et al., 2020; Eichenlaub et al., 2021).

Noninvasive mapping comprises solving the inverse problem of electrocardiography (i.e., calculating the cardiac electrical activity based on the body surface potentials) (Cuppen et al., 1984). However, solving the inverse problem of electrocardiography is not easy, because it is essentially ill-posed (i.e., multiple, very different solutions can explain the same ECG). As a consequence, various assumptions have to be made and physiological knowledge has to be added to the equations in order to select a plausible solution.

Solving the inverse problem depends on the choice of the cardiac source model to describe the cardiac electrical activity (Van Oosterom, 2014). In this paper, we describe the two most commonly used distinct cardiac source models used in inverse methods; the epicardial potential model and the equivalent dipole layer model. We discuss similarities and differences in applicability, strengths and weaknesses and sketch a road towards improved inverse solutions by a combination of the two methods.

## 2 Description

The actual current source that generates the ECG is the current that flows over the myocardial membrane. The direction and strength of this current depends on the gradient of the transmembrane potential within the myocardium (Plonsey and Barr, 1987; Van Oosterom, 2014):

$${\vec{J}}^{i}\left(\vec{r}\right)=-\sigma_{i}\vec{\nabla }\varphi_{m}\left(\vec{r}\right)$$ (1)

With ${\vec{J}}^{i}\left(\vec{r}\right)$ the impressed current density at location $\vec{r}$ within the myocardium, $\sigma_{i}$ the electric conductivity of the intracellular medium and $\varphi_{m}\left(\vec{r}\right)$ the transmembrane potential at $\vec{r}$ .

The potentials generated in a volume conductor (such as the body) by the volume current source distributions inside the heart are exactly the same as those generated by an equivalent surface source distribution at a surface that encloses all active sources, such as the epicardium (Barr et al., 1977). From this it follows that the actual current sources *within* the myocardium cannot be determined from potential recordings outside the heart, but an equivalent source *at the surface* of the heart can.

Two different equivalent surface source models are used in inverse electrocardiography: the Epicardial Potential (EP) source model, and the Equivalent Dipole Layer (EDL) source model.

### 2.1 Epicardial potential source model

**FIGURE 1:**
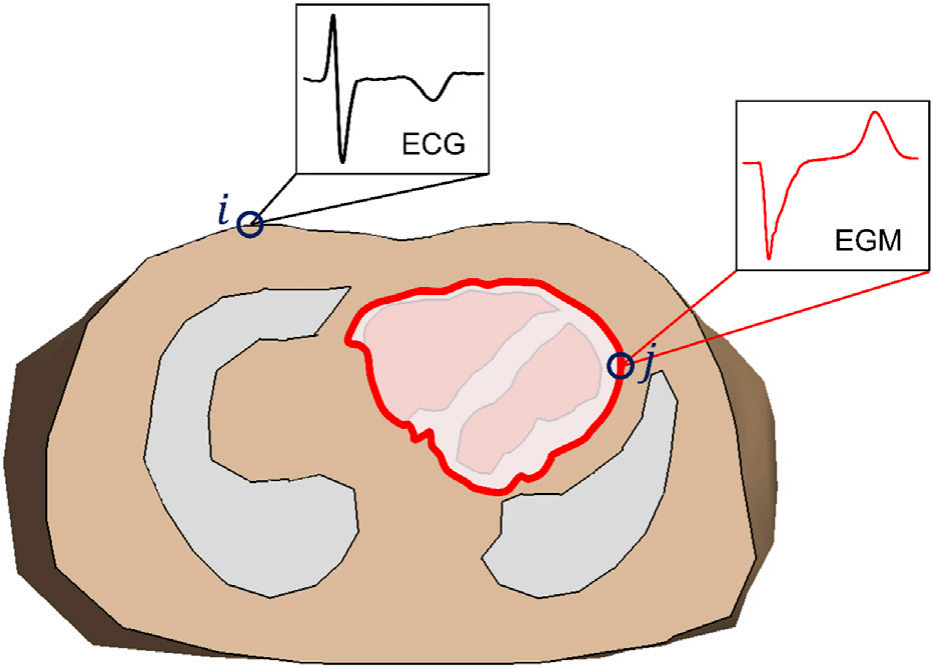
Transversal cross section of a volume conductor model of the thorax, showing lungs and ventricles. The red line indicates the location of the epicardial potential (EP) source (in this example limited to the ventricles): a closed surface that contains all electric sources within the ventricular myocardium. In the volume conductor model for the EP-based inverse method the source surface is considered as an internal boundary of the volume conductor; all tissue within that surface is ignored. The transfer matrix describes the relation between the electrograms (EGM) at the epicardial surface (j) and the electrocardiogram (ECG) at the body surface (i).

In the EP source model, the current sources within the myocardium are replaced by an internal boundary of the volume conductor that encompasses the heart, at which the same potentials are impressed as those that are generated by the actual sources (Figure 1). Using a volume conductor model (see Supplementary Material for details), the transfer matrix T can be calculated that relates $V_{i}\left(t\right),$ the extracellular potential at electrode i at body surface at time t , to the potentials $\varphi_{j}\left(t\right)$ at that moment at all $N_{h}$ discretization nodes of the myocardial surface that encompasses the heart:

$$V_{i}\left(t\right)=\sum_{j=1}^{N_{h}}T_{ij}\varphi_{j}\left(t\right)\text{or in vector form}V\left(t\right)=T\varphi \left(t\right)$$ (2)

In this equation, $T_{ij}$ (element i,j of matrix T ) is the potential at electrode i if at the epicardium node j has potential 1, and all other nodes have potential 0. T is an $N_{e}\times N_{h}$ matrix, with $N_{e}$ the number of electrodes on the body surface and $N_{h}$ the number of nodes on the myocardial surface. $V\left(t\right)$ and $\varphi \left(t\right)$ denote vectors with respectively elements $V_{i}\left(t\right)$ for $i=1\ldots N_{e}$ and $\varphi_{j}\left(t\right)$ for $j=1\ldots N_{h}$ .

One might expect that a simple matrix inversion would now produce the epicardial potentials (electrograms) from the recording ECGs, but that is not the case. First of all, matrix T is singular if the number of body electrodes is smaller than the number of heart nodes, which generally is the case. But even for a large enough number of electrodes, the inverse problem remains *ill posed*: very different potential distributions at the heart will give rise to almost identical ECGs on the body surface. Because of this, noise in the recording would result in estimates of epicardial potentials that are very different from the true epicardial potentials.

This problem is commonly overcome by regularization (Gulrajani, 1998). This involves adding additional constraints to the solution, for instance that small (zero-order Tikhonov) or smooth (second order Tikhonov) epicardial potentials are preferred. For zero-order Tikhonov regularization the inverse problem for the EP source model reads: for each sample time t , find the epicardial potentials $\varphi \left(t\right)$ that minimizes

$$\left\|V\left(t\right)-T\varphi \left(t\right)\right\|+\lambda \left\|\varphi \left(t\right)\right\|$$ (3)

where λ is the regularization parameter that determines the relative weight of the additional constraint. Finding the optimal value for λ , i.e., the value that gives a solution that best matches the true epicardial potentials, is not an easy task. Popular strategies are zero-crossing (Johnston and Gulrajani, 1997), L-curve methods (Hansen and O’Leary, 1993) and Composite REsidual and Smoothing Operator (CRESO) (Colli-Franzone et al., 1985). Their definitions and differences are outside the scope of this paper, but have been discussed by others (Milanič et al., 2014; Chamorro-Servent et al., 2019). An extensive overview of different mathematical techniques to solve the inverse problem for the EP method is given by Borràs and Chamorro-Servent (2021).

### 2.2 Equivalent dipole layer source model

**FIGURE 2:**
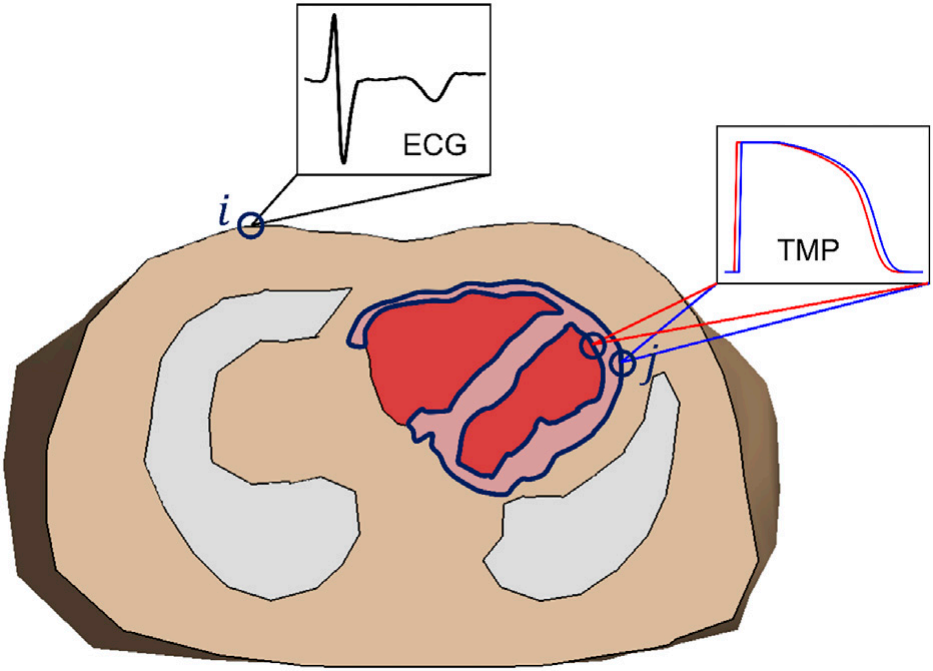
Transversal cross section of a volume conductor model of the thorax, showing lungs and ventricles, and intraventricular blood mass. The dark blue line indicates the location of the equivalent dipole layer source (in this example limited to the ventricles): the surface of the myocardial tissue. Notice that this includes both epicardium and endocardium. The transfer matrix describes the relation between the transmembrane potential (TMP) at the endo-/epicardial surface (j) and the electrocardiogram (ECG) at the body surface (i).

Geselowitz showed that under certain assumptions, the body surface ECG generated by the actual source activity within the myocardium is equal to the ECG generated by a layer at the surface of the myocardium (Figure 2, blue line) that injects current perpendicular to the surface (a dipole layer) (Geselowitz, 1992). The strength of the injected current in this model is proportional to the local transmembrane potential.

Similar to the EP-based method, a volume conductor model is used to compute the transfer matrix that relates the potential at ECG electrodes to the dipole layer strength (and hence the transmembrane potential) at the boundary delineating the entire myocardial surface (Huiskamp et al., 1988; Van Dam et al., 2009):

$$V_{i}\left(t\right)=\sum_{j=1}^{N_{h}}A_{ij}d_{j}\left(t\right)\text{or in vector form}V\left(t\right)=Ad\left(t\right)$$ (4)

with $A_{ij}$ the transfer matrix, i.e., the potential generated at body electrode i by a unit-strength dipole layer element at source node j , and $d_{j}\left(t\right)$ the dipole layer strength at that node at time t . Note the difference in nature of the transfer matrices T and A for, respectively, the EP and EDL methods. T expresses the effect of impressed potential at the epicardial boundary, whereas A expresses the effect of impressed current at the myocardial surface (both endo and epicardial boundary). In Section 3.3 we will discuss the consequences of this difference.

**FIGURE 3:**
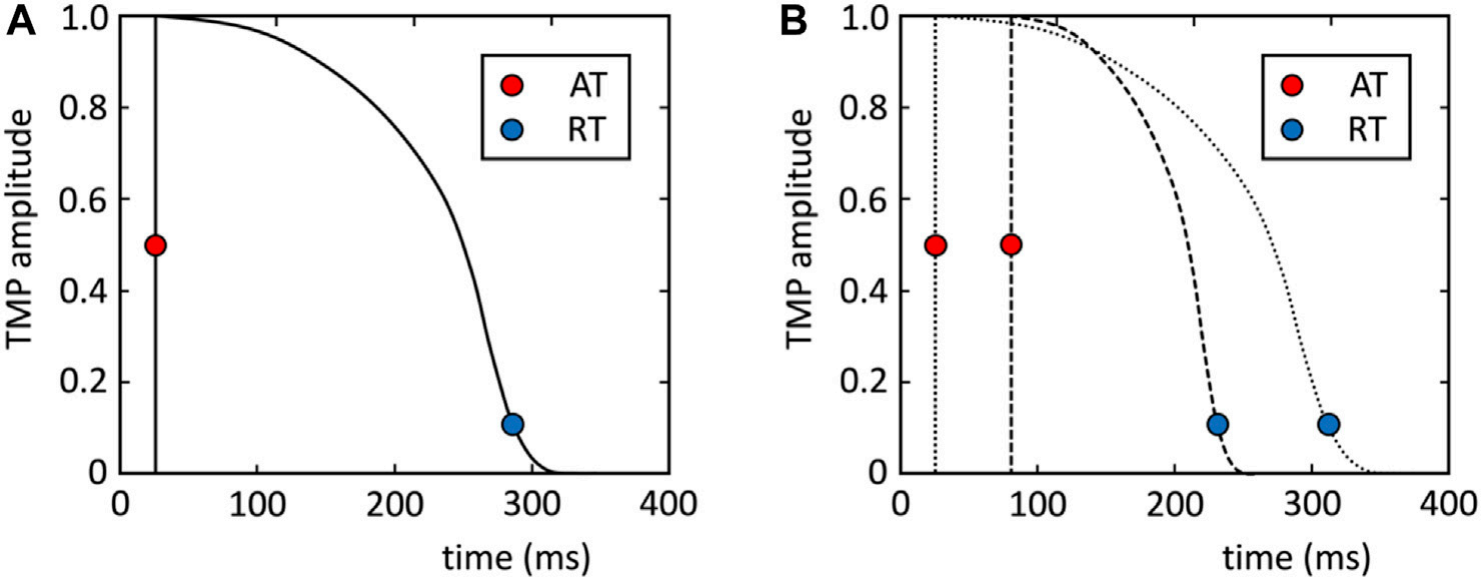
**(A)** Template for transmembrane potential (TMP) (see Supplementary Material). **(B)** This template is shifted and stretched to match the activation and repolarization times (AT and RT) of the nodes at the myocardial surface (example for two nodes are plotted).

Theoretically, Eq. 4 would allow the estimation of the transmembrane potentials at the endo- and epicardium at each moment in time, although it turns out to be too ill-posed. Instead, the EDL-based inverse uses a template for the transmembrane potential that is based on the T-wave of the recorded ECGs (Van Oosterom, 2004; Van Dam et al., 2009); see Supplementary Material for details. For each source node j , this template is shifted and stretched to match the activation time $\tau_{j}$ and repolarization time $\rho_{j}$ of that node (see Figure 3), resulting in a dipole layer waveform per node that depends on the timing of that node: $d\left(\tau_{j},\rho_{j},t\right)$ . In vector form we write the dipole layer waveform for all nodes at the myocardial surface as $d\left(\tau ,\rho ,t\right)$ , with τ and ρ the vectors representing the activation and repolarization times of all nodes. The relation between the timing at the heart surface and the potential at the body surface then reads:

$$V\left(t\right)=A\;d\left(\tau ,\rho ,t\right)$$ (5)

The EDL-based inverse method solves the activation and repolarization times by minimizing the difference between the recorded potentials and the modeled potentials from Equation 5 along the complete QRST interval. As this problem is also ill posed, regularization is needed for the EDL-based inverse as well. The Laplacian of the activation and repolarization times is used as regularization operator, thus preferring smooth activation and repolarization patterns. The EDL-based inverse then reads: for all electrodes i and all myocardial surface nodes j , find $\tau_{j}$ and $\rho_{j}$ that minimize for all sample times simultaneously:

$$\left\|V\left(t\right)-A\;d\left(\tau ,\rho ,t\right)\right\|+\lambda_{\tau }\left\|L\left(\tau \right)\right\|+\lambda_{\rho }\left\|L\left(\rho \right)\right\|$$ (6)

with L the operator that computes the L2 norm of the Laplacian, and $\lambda_{\tau }$ and $\lambda_{\rho }$ regularization parameters. Similar to the EP-based method, finding the optimal $\lambda_{\tau }$ and $\lambda_{\rho }$ regularization parameters is not straightforward. Approaches involve choosing an empirically determined fixed value (Van Dam et al., 2009) or varying the parameter to aim for an empirically defined value for the Laplacian operator, indicating a certain spatial smoothness (Van der Waal et al., 2021).

In contrast to the EP-based inverse, the EDL-based inverse is a non-linear problem. The Marquardt procedure (Marquardt, 1963) is used to solve this problem by iteration from an initial estimate of the activation and repolarization times. The initial estimate of the activation times is obtained by creating activation patterns from all heart nodes, using the shortest route algorithm, and selecting the one for which the potentials according to Equation 5 best match the recording potentials (Van Dam et al., 2009). Additional starting points are added for ECGs not resulting from a premature ventricular complex (PVC). The initial estimate of repolarization is calculated from that of activation, assuming that early activated sites have longer action potential duration (APD) than late activated sites, based on myocardial electrotonic interaction (Van Dam et al., 2009), although more recently it has been suggested that a uniform APD might be better for ventricular paced activations (Van der Waal et al., 2022).

## 3 Differences between EP- and EDL-based inverse methods

The EP- and EDL-based methods can both estimate electrophysiological parameters from recorded ECGs. However, they do this is a very different way, and both methods have their strong points and weak points. Table 1 summarizes the differences between the two methods.

**TABLE 1:** Description of different aspects of the two cardiac source models for inverse electrocardiography.

	Epicardial Potential	Equivalent Dipole Layer
Source model characteristics		
Type of source model	Epicardial electrogram = impressed potential at epicardium	Current dipole layer at myocardial surface (epicardium and endocardium) = impressed transmembrane potential at myocardial boundary
Model parameter	Direct: Local electrogramDerived; AT/RT	Direct: AT/RT Derived: local electrogram Potse et al. (2009)
Volume conductor model	Only epicardial surface; All tissue within epicardium is ignored	Epicardial and endocardial surface
Imaging possibilities	Only epicardial surface–any imaging modality	Endocardial surface–needs MRI or CT with contrast
Describes sources *within* the myocardium	No	No
Dimensionality	N_p_ x N_t_: Potential at N_p_ locations at the epicardial surface at N_t_ time instances	2 x N_p_: Activation and repolarization times at N_p_ locations at epi- and endocardium
Assumptions	None	1. Equal anisotropy ratio of intra- and extracellular medium 2. TMP waveform at surface follows a single template, with T_dep_ and T_rep_ as parameters
Linearity	linear: “simple” matrix inversion	non-linear: non-linear parameter estimation; initial estimate required
Sensitivity to ill-posedness	High	Intermediate
Regularization	Physiologically unrealistic: smallest/smoothed solution not best solution	Physiologically realistic: smooth activation pattern is realistic
Source model applications		
Validated for activation/repolarization	Yes/yes (Table 2)	Yes/yes (Table 2)
Analyzable rhythms	Sinus rhythm, PVC, VT, VF	Sinus rhythm, PVC (needs distinguishable QRS and T wave on ECG)
Phase-analysis during VT/VF	Yes	No
Localization of endocardial focus	Derived from breakthrough characteristics at epicardium	Directly
Localization of septal focus	No	Directly
Can be used with structural abnormalities	Yes (but see note in Section 3.6)	Yes (myocardial infarction, Section 3.6)

AT, activation time; RT, repolarization time; TMP, transmembrane potential; PVC, premature ventricular complex; VT, ventricular tachycardia; VF, ventricular fibrillation.

### 3.1 Assumptions

The EP-based method concentrates the potentials generated by the heart sources on a surface that encompasses the heart. This surface is just outside the epicardium. Therefore, the reconstructed potentials rather should be considered as the potentials that would have been measured in close proximity to, but not on the heart.

In the EDL-based method, the equivalence of the EDL and the actual sources within the myocardium is only true if anisotropy ratios of intracellular and extracellular conductivity myocardial are equal (Geselowitz, 1992). In reality, this is not the case. A model study has shown that this leads to an root-mean-square error in estimated activation times of 15–20 ms (Janssen et al., 2018). A second assumption for the EDL-based method is that the TMP waveform at the surface follows a single template, with activation and repolarization times as parameters. However, it is known that the shape of the TMP shows regional differences in the heart, mainly between endocardium and epicardium, due to different properties of the transient outward current (Nabauer et al., 1996). In addition, acute myocardial ischemia can alter the amplitude by influencing the resting membrane potential (Janse and Wit, 1989). Incorporation of a variable TMP may improve the accuracy of the method and is topic for further exploration.

Since the inverse problem is ill posed, both inverse methods require regularization to prevent very unphysiological solutions driven by measurement noise. The choice of the regularization parameter determines to what extent the effect of noise is reduced by forcing the solution to comply with *a priori* assumptions.

With the EP-based method, regularization introduces the implicit assumption that epicardial potentials are either small (zero order Tikhonov) or smooth (second order Tikhonov). Both assumptions are inaccurate; there is a large gradient in epicardial potentials at the edge of the activation wavefront on the epicardium. This regularization tends to result in epicardial potentials that are much smaller and smoother at the wave front than what is recorded in electrograms. EP-based validation studies indeed report epicardial electrograms that are smaller in amplitude than recorded electrograms (Bear et al., 2018a), and are mostly incorrect in regions with changing electrogram morphologies (Cluitmans et al., 2017a), which obscures the detection of electrical heterogeneities such as caused by myocardial infarctions.

In the EDL inverse, regularization operates on activation and repolarization times. In the EDL-based method, the implicit assumption in second order Tikhonov regularization is that activation and repolarization patterns are smooth; this is physiologically realistic. The values of regularization parameters are chosen by demanding that the result of the Laplacian operator, and hence the amount of smoothness, is a value that corresponds to that of realistic activation and repolarization patterns. In pathological conditions (e.g., myocardial infarction and arrhythmogenic syndromes) electrical heterogeneities may be present in the heart. In such cases the amount of regularization required to suppress the effect of noise may obscure the presence heterogeneities in the EDL based inverse. It has been shown that a large area of repolarization heterogeneity is accurately inversely reconstructed (Van der Waal et al., 2021), but for smaller areas of heterogeneity this is unknown.

### 3.2 Calculation of activation and repolarization times

The direct outcome of the EP-based inverse method is the epicardial potential distribution. From this, activation and repolarization times (AT and RT, respectively) can be calculated in the same way as they are derived from electrograms, by determining the time of maximum downward slope during activation and that of the maximum upward slope during repolarization (Haws and Lux, 1990; Coronel et al., 2006). This potentially introduces errors into the EP-based solution, since the accuracy of these values is reduced by the smearing effect of regularization. In addition, it has been demonstrated that falsely fractionated (reconstructed) electrograms lead to incorrect ATs in certain areas of the heart (Bear et al., 2019a) and can even lead to artificial lines of block (Duchateau et al., 2019). Improvements in accuracy have been presented when using a spatiotemporal filter for AT/RT detection from electrograms (Duchateau et al., 2017; Cluitmans et al., 2022), although this introduces additional filtering over the surface, which is similar to the smoothing that is applied in the EDL method.

The EDL-based inverse method estimates the activation and repolarization times directly from the recorded ECGs. Note that the EDL repolarization time is linked to the transmembrane potential (TMP) at that particular location, whereas the EP- and electrogram-based repolarization times are determined from epicardial potentials that are the result of currents generated in a larger volume. Experimental and model studies have shown the correspondence of epicardial potential slope and TMP repolarization timing (Coronel et al., 2006; Potse et al., 2009), confirming the ability of using this to determine accuracy of the EDL-based inverse method.

### 3.3 Endocardial activity

In most implementations of the EP source model, a surface surrounding the outside of the heart is chosen as the EP source surface, as in Figure 1. Consequently, the potentials at the endocardium are not reconstructed by the EP inverse. In contrast, the source surface of the EDL-based inverse includes both the epicardium and endocardium (see Figure 2), allowing the estimation of source activity everywhere on the myocardial surface.

The source surface for the EP-based inverse method can be chosen equal to that of the EDL-based method, but there is little use in that: the epicardial surface almost surrounds that of the endocardium (except at the most basal parts of the heart), and, consequently, influence of endocardial potentials is almost completely shielded by epicardial potentials. As a result, elements of the transfer matrix T are very small for endocardial nodes. In the EDL-based method, on the other hand, the equivalent source is a current source. Current generated at the endocardium is not blocked by the epicardial part of the equivalent source, but the currents from these two parts add up. Consequently, the EDL-based method finds activation and repolarization times at both endocardium and epicardium by necessity.

This difference between the EP and EDL-based methods can be visualized by constructing sensitivity maps, as introduced by Huiskamp et al. (1988) for the EDL source model (Van Oosterom and Huiskamp, 1989). The elements of the sensitivity matrix $S_{A,ij}$ for the EDL source model is defined as

$$S_{A,ij}=A_{ij}/\alpha_{j}$$ (7)

with $\alpha_{j}$ the surface area of discretization element j at the source surface. $A_{ij}$ may be interpreted as the sensitivity of electrode i to source activity at source element j (the division by $\alpha_{j}$ is required in order to correct for the difference in size of source elements). Consequently, a map on the heart of row i of $S_{A}$ visualizes the sensitivity of electrode i to source activity in different regions of the heart, the sensitivity map of electrode i . The sensitivity matrix $S_{T}$ for the EP source model is constructed in the same way from the corresponding transfer matrix T .

**FIGURE 4:**
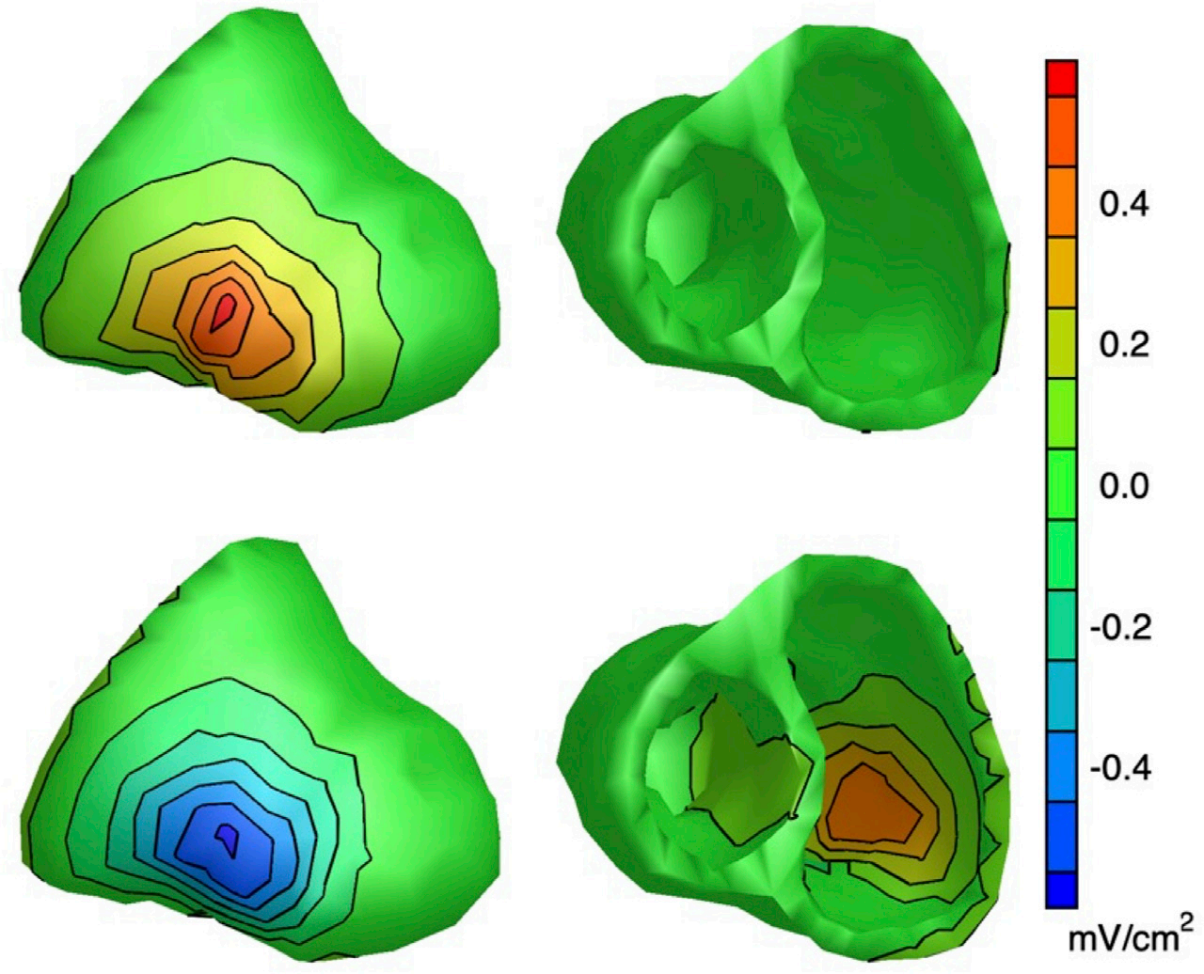
Lead V2 sensitivity maps: maps of the sensitivity of electrode V2 to source activity at the heart surface [see Van Oosterom and Huiskamp (1989) for details]. Top row: EP sensitivity map for the EP method; source activity is defined as a region of 1 cm^2^ that impresses 15 mV at the location considered, and zero elsewhere. Bottom row: sensitivity map for the EDL method; source activity is defined as a completely depolarized 1 cm^2^ region at the location considered, and complete polarization elsewhere. For the EP method, the strongest sensitivity for the epicardium is 0.57 mV/cm^2^ (at the location closest to V2), whereas the strongest sensitivity for the endocardium is -0.0019 mV/cm^2^. For the EDL methods, these values are -0.56 mV/cm^2^ and 0.43 mV/cm^2^ respectively. This shows that the EP-based inverse, in contrast to the EDL-based inverse, is completely insensitive to the endocardium.

The top row of Figure 4 shows the EP sensitivity map of lead V2, for a source surface that encompasses both the endocardium and the epicardium. It is expressed as the contribution in mV to the ECG in lead V2 by 15 mV impressed potential at 1 cm^2^ of the surface. The value of 15 mV was chosen because this gives the same maximum contribution from the epicardium to lead V2 as the EDL does; it is also a realistic value for the epicardial potential during depolarization. The figure demonstrates that in the EP source model the surface ECG is only sensitive to the epicardial part of the source surface.

The bottom row of Figure 4 displays the EDL sensitivity map of lead V2. Note that, as expected, the contribution of the epicardium of the left free wall to lead V2 is negative (activity at this location only is equivalent to an inward moving activation wave front from that location), and that of the left free wall endocardium is positive. The contribution of the endocardium of the left free wall is somewhat smaller than that of the epicardium (because of the larger distance to lead V2), but still considerable. This demonstrates that in the EDL source model V2 is sensitive to both the epicardium and the endocardium.

Wang et al. (2018) used the EP-based inverse method with a source surface that includes the endocardium, like in Figure 4. Their results show non-zero potentials at the endocardium, most likely because they used second order Tikhonov regularization, which requires the solution to be smooth. In regions where contribution to the surface ECG is small, this results in potentials being determined almost completely by extrapolation between regions that do contribute (Oostendorp et al., 1989).

### 3.4 Initial estimate

As the EP-based inverse method is a linear problem, it has a unique solution. The epicardial potentials that minimize Eq. 3 can be easily determined, without the need for an initial estimate.

In contrast, the EDL-based inverse is a non-linear problem, and requires a first estimate of activation and repolarization times, and from there the solution is optimized in iterative steps. This is analogous to finding a route downhill in a mountainous region: starting at different locations one may end up in different valleys. In the EDL context, this means that different initial estimates may result in very different reconstructed activation and repolarization patterns. This implies that the initial estimate needs to be as close as possible to the actual pattern.

In an EDL-based simulation study (Janssen et al., 2018), the reconstructed activation patterns were close to the actual patterns in most cases, but there were a few cases in which they were grossly inaccurate. In those cases, the match between the reconstructed and recorded ECGs was also worse. Closer inspection of the initial estimate in those cases revealed that there were two distinct initial estimates, for which the match between the reconstructed and recorded ECGs was almost equal. The initial estimate that had a slightly better match with the body surface ECG resulted in an erroneous final solution after optimization. In these cases, the other initial estimate produced a markedly improved final solution. As a general strategy, starting from several initial estimates and then taking the final solution that produces the best fit for the ECGs might be a solution to improve the stability. As an alternative, the different solutions can be presented to the practitioner, thus making the uncertainty in the inverse explicit.

### 3.5 Accuracy

Even though the technique has been used for decades and has already been adopted in commercially available systems [CardioInsight™ (Medtronic), Amycard (EP Solutions), Acorys (Corify Care)], validation studies are relatively scarce. We believe that this is due to the difficulty in obtaining the gold standard [i.e., epicardial (and endocardial) electrograms and corresponding activation and repolarization timings], especially in human studies. Validation can be achieved by the accuracy of localization of an ectopic focal activity, of the activation or repolarization pattern, or of the localization of an arrhythmogenic substrate. An overview of studies investigating the accuracy under various conditions is given in Table 2. Most studies report correlation coefficients of >0.60, indicating a good fit. The pacing localization error is a relatively well-defined measure for both inverse source models, ranging from ∼5 to 30 mm with EP-based and from ∼0 to 25 mm with EDL-based method. However, this table illustrates many different subject conditions for the validation studies, differences in analyzed rhythms, and also differences in reporting outcomes. Errors are usually reported as a mean absolute error, but a root-mean-square error is also used in some studies. Correlation coefficients (CC) are usually reported as medians with a CC for each activation/repolarization pattern of the reconstructed beat, although one study also pooled all beats together to calculate one single CC (Cluitmans et al., 2017a). These differences make it difficult to compare accuracy results within the same source model, but especially when attempting to compare between the two source models. Studies comparing the accuracy of the two cardiac source models are limited to simulated data (Cheng et al., 2003; Van Oosterom, 2014), which showed a higher correlation for EDL than EP-based method. For a more detailed comparison of the accuracy of the two cardiac source models, both methods need to be applied to the same physiological dataset.

**TABLE 2:** Summary of relevant validation studies using the Epicardial Potential or Equivalent Dipole Layer cardiac source model for inverse electrocardiography.

Epicardial potential source model											
Method	Species	Subject conditions	#sub-jects	# beats	Rhythms	Electrogram correlation	Pacing loc. error (mm)	AT error (ms)	AT CC	RT error (ms)	RT CC
*Ex-vivo* Bear et al. (2021)	Pig	Normal & electrical (RT) heterogeneities	8	24	Sinus	0.85 [0.52–0.96]	-	-	-	25 [19–31]	0.73 [0.63–0.83]
*Ex-vivo* Bear et al. (2019b)	Pig	Electrical (RT) heterogeneities	3	55	Pacing from atria, LV&RV epi	-	-	7.8 ± 3.2 RMSE	0.86 ± 0.11	17.4 ± 3.6 RMSE	0.83 ± 0.13
*Ex-vivo* Bear et al. (2018b)	Pig	LBBB	11	39	Sinus (17) and LV (7) & RV (8) & BiV (7) epi pacing	-	9.1 ± 0.6^a^	13.4 ± 5.3 RMSE	0.68 ± 0.25	-	-
*Ex-vivo* Bear et al. (2019a)	Pig	LBBB & electrical (RT) heterogeneities	8	AT 8, RT 12	Sinus	-	-	7.5 ± 4.3	0.75 ± 0.13	28 ± 11	0.64 ± 0.16
*In-vivo* Bear et al. (2018a)	Pig	Normal	5	70	Sinus and LV&RV endo&epi pacing	0.72 [0.40–0.87]	16 [9–29]	-	0.78 [0.70–0.81]	-	-
*In-vivo* Bear et al. (2021)	Pig	Normal	5	∼90	Sinus and endo&epi paced	0.86 [0.52–0.96]	-	-	-	10 [8–13]	0.76 [0.67–0.82]
*In-vivo* Hohmann et al. (2019)	Pig	Normal	9	118	LA,RA,LV, RV endo (109) & epi (9) pacing	-	21 [13–29]	-	-	-	-
*In-vivo* Cluitmans et al. (2017a)	Canine	Normal	4	93	Atrial (13) LV&RV endo (5) & epi (71), or BiV (4) pacing	0.71 [0.36–0.86]	10 [7–17]	-	0.82	-	0.73
*In-vivo* ^b^ Ghanem et al. (2005)	Human	MI, AF, aortic aneurysm	3	5	Sinus & RV endoandepi pacing	0.72 ± 0.25	∼10	-	-	-	-
*In-vivo* Sapp et al. (2012)	Human	VT ablation, structural abnormalities?	4	79	Epi pacing	-	13 ± 9	-	-	-	-
*In-vivo* Duchateau et al. (2019)	Human	Substrates: BrS, ARVC, DCM, EarlyRep or Idiopathic VF	55	59	Sinus (53) & RVendo pacing (6)	-	-	20.4 ± 8.6	0.03 ± 0.43	-	-
*In-vivo* Graham et al. (2019)	Human	Substrates: ARVC, IHD, BrS, DCM	8	8	Atrial (1), RV (6) and BiV (1) pacing	0.65 [0.71–0.74]	21 [10–33]	24 [21–35] RMSE	0.66 [0.53–0.73]	51 [38–70] RMSE	0.55 [0.41–0.72]
Equivalent Dipole Layer source model											
Method	Species	Subject conditions	#sub-jects	# beats	Rhythms	Correct endo/epi localization	Pacing loc. error (mm)	AT error (ms)	AT CC	RT error (ms)	RT CC
*Ex-vivo* Oosterhoff et al. (2016)	Pig	Normal	2	4	2 sinus, 2 PVC (spontaneous)	*-*	0 and 5 mm	8.4 ± 6.4	-	-	-
*Ex-vivo* Van der Waal et al. (2021)	Pig	Normal & electrical (RT) heterogeneities	4	20	Atrial, LV&RV epi paced	-	-	∼13	-	26 ± 22	0.74 baseline, 0.63 all conditions
*In-vivo* Oosterhoff et al. (2016)	Pig	Normal	4	29	Sinus (2) and endo (15) & epi (12) paced	85% (27% of endo incorrect epi)	18 [15–27]	11 ± 5 & 5 ± 3 (sinus only)	0.53 & 0.82 (sinus only)	-	-
*In-vivo* Stevenson et al. (1993)	Human	WPW patients	7	7	Sinus	-	19 ± 6^b^	-	-	-	-
*In-vivo* Roudijk et al. (2021)	Human	Cardiomyopathy	13	13	Sinus	-	-	17 ± 7 epi, 20 ± 8 RVendo, 28 ± 9 LVendo	0.54 ± 0.19 epi, 0.50 ± 0.27 RVendo, 0.44 ± 0.29 LVendo	-	-
*In-vivo* Boonstra et al. (2022)	Human	Cardiomyopathy	4	4	Sinus	-	-	14 [9–25] epi, 20 [10–30] endo	0.64 [0.41–0.91] epi, 0.54 [0.19–0.81] endo	-	-

^a^ Localization error of latest moment of activation, ^+^ Non-simultaneous recording.

^b^ Detection of accessory pathway insertion site.

AT, activation time; RT, repolarization time; CC, correlation coefficient; LV, left ventricle; RV, right ventricle; RMSE, root mean square error; LBBB, left bundle branch block, endo, endocardium, epi, epicardium, LA, left atrium; RA, right atrium, BiV, biventricular, MI, myocardial infarction; AF, atrial fibrillation; VT, ventricular tachychardia, BrS, brugada syndrome, ARVC, arrhythmogenic right ventricular cardiomyopathy; DCM, dilated cardiomyopathy, EarlyRep, Early repolarization syndrome, VF, ventricular fibrillation; IHD, Ischemic heart disease. Presented timing differences are mean absolute errors, unless stated otherwise. Median [IQR] or mean ± SD.

### 3.6 Myocardial scar tissue

In many cardiac patients regional and intramural fibrotic zones and scars are present, for instance as the result of myocardial infarction, inflammation or the Brugada syndrome. Within these regions, that may differ in extent and heterogeneity between patients, electrically inexcitable pathways may remain (Stevenson et al., 1993). This has different consequences for the EP and EDL source models, as described below.

In a world free of noise and free of modeling errors, the EP inverse would reconstruct the actual epicardial potential, irrespective of whatever is inside the epicardium. Scar tissue will lead to epicardial potentials that are lower in amplitude, and to fractionation in electrograms. These aspects can be reconstructed with the EP-based inverse method, which can be used to identify the “electrical scar” (Cuculich et al., 2011). However, it is also noted that the presence of nearby scar significantly reduced the accuracy of the pacing localization with the EP-based method (Sapp et al., 2012). Moreover, the use of regularization most likely affects the reconstruction of lower amplitude and fractionated electrograms.

In the EDL-based method, the equivalent source surface is the boundary of the myocardial tissue that participates in electric activity of the heart. In case of scar tissue, where necrotic/fibrotic tissue is unexcitable, the endocardial and epicardial surfaces together do not constitute a correct equivalent source surface. It has been suggested that old myocardial infarctions can be modeled by defining the surface of all viable myocardial tissue as the location of the equivalent source. This requires creating a hole through the myocardium at the infarct location (Oostendorp et al., 2002). Parameters that are reconstructed with the EDL-based method are activation and repolarization time. Therefore, regions of slowed conduction or inhomogeneous activation, which is the cause for broad fractionated electrograms (Gardner et al., 1985), may be detectable with this method. However, smoothing induced by regularization of EDL-based solutions may mask these small inhomogeneities.

In comparison, the fact that the EP source model must ignore the electric properties of the volume inside the epicardium is both a blessing and a curse: there is no need to adapt the source model in the case of scar tissue, but also there is no easy way to explicitly include prior information such as presence of unexcitable tissue, which may improve the solution to this ill-posed problem.

### 3.7 Arrhythmia mapping

With the EP-based inverse, reconstruction of electrograms during an arrhythmia is also possible. This allows analysis of episodes of arrhythmia by phase mapping to detect rotors, to indirectly determine cycle length and mechanism (focal or reentry) (Umapathy et al., 2010; Haïssaguerre et al., 2018). This does require a controlled setting, since body surface ECG recordings during spontaneous arrhythmia are rare.

The current implementation of the EDL-based inverse method uses a template for the transmembrane potential during a cardiac cycle. It therefore cannot cope with a second cycle starting while the first has not yet finished everywhere within the myocardium. However, this method may be useful to find potential sites of reentry noninvasively, even if a reentry does not actually occur. A cardiac map of the reentry vulnerability index, an activation-repolarization time metric that is a measure for reentry vulnerability, can be constructed from the activation and repolarization map of subsequent beats (Child et al., 2015; Orini et al., 2020; Jelvehgaran et al., 2023). This can also be derived from timing maps obtained by the EP-based method, but those do not include the endocardium.

## 4 Combining inverse methods

In the previous section we have shown that the EP- and EDL-based inverse methods both have their strengths and weaknesses. This begs for a procedure that combines the strong points of each method. We identified two ways in which this might be achieved:

• *Sequential.* A weak point of the EDL is that it requires an initial estimate close to the optimal solution. Activation and repolarization times obtained by the EP method may be used to provide an initial estimate for the epicardial timing values. An initial estimate is then still required for the endocardial timing; this can be obtained with the existing initial estimation method while keeping the epicardial values fixed at those provided by the EP method. The main advantage over using only the EP method is that in this way also the endocardial timing is estimated.
• *Merging.* The two methods can be combined into a single non-linear estimation procedure, that minimizes Eqs 3 and 6 simultaneously. One way of achieving that is to use the EP method to obtain a first solution and determine activation and repolarization for these estimated electrograms. Subsequently, estimated ECGs can be computed for both the EP and EDL method, and a solution of the epicardial potentials can be found iteratively that minimizes the error for both estimates simultaneously:

$$\left\|V\left(t\right)-T\varphi \left(t\right)\right\|+\lambda \left\|\varphi \left(t\right)\right\|+\left\|V\left(t\right)-A\;d\left(\tau ,\rho ,t\right)\right\|+\lambda_{\tau }\left\|L\left(\tau \right)\right\|+\lambda_{\rho }\left\|L\left(\rho \right)\right\|$$ (8)

In such a merging procedure it needs to be considered how to handle regularization. It would be possible to maintain both regularization methods, each with their own regularization parameter, or choose only one.

These techniques have not been implemented and tested before, so we recommend further research into this to determine accuracy and feasibility. To be fair, successful implementation of such a combined inverse is not assured. Some issues that may need to be overcome are:

• The merged inverse is a non-linear problem, and needs to be solved by non-linear parameter estimation. Conversion is not assured; it may depend strongly on the quality of the initial estimate.
• Finding an optimal value for a single regularization parameter is already not so simple, finding the combined optimum for three regularization parameters might proof very complicated.
• There is a risk that the complexity of this model leads to overfitting.

## 5 Discussion

After being introduced in the 1970s (Martin and Pilkington, 1972), the EP-based inverse method has gained a lot of attention, leading to many scientific studies to apply and improve the method, by quantifying and overcoming issues with for example, regularization (Milanič et al., 2014; Cluitmans et al., 2017b; Chamorro-Servent et al., 2019), geometric inaccuracies (MacLeod et al., 2000; Cluitmans and Volders, 2017; Tate et al., 2021) and spatial filtering (Duchateau et al., 2017; Cluitmans et al., 2022; Schuler et al., 2022). The development of a commercial system also increased the popularity of this method.

The introduction of the EDL as a source model for the inverse problem was first documented in 1984 (Cuppen et al., 1984), and although it also gained attention, research studies into technical difficulties involving this method are not as numerous as for the EP-based method. Further research into some of the difficulties of the EDL-based inverse as discussed above (e.g., the initial estimate, regularization, application of the method to structurally abnormal hearts) might provide valuable insights and improve accuracy (and therefore, clinical applicability).

A midmyocardial layer of M-cells was not included in the initial estimate of the EDL-method. The reason for this is that a closed intramural dipole layer, by definition, does not generate an equivalent epicardial or endocardial dipole layer (Figure 2). The EDL-method therefore generates only an activation and repolarization estimation on these surfaces and not intramurally. In addition, the M-cells likely do not play a large role in intact and *in vivo* hearts (Opthof et al., 2016).

One potential source of inaccuracies that applies to both methods lies in the volume conductor models. In both methods, these models are assumed static; the effect of breathing and cardiac motion are not taken into account. The latter may be of influence for repolarization mapping. Although this is briefly mentioned as a possible source of error in many papers (MacLeod et al., 2000; Cluitmans et al., 2018; Bear et al., 2021), and is quantified to be correlated to accuracy (Jiang et al., 2009; Cluitmans et al., 2017a), it is not commonly incorporated into the inverse ECG method. The implementation of a dynamic volume conductor model could potentially contribute to improving both methods.

The fact that 100% accuracy can never be reached in inverse ECG should be taken into account by the interpreting clinical physician. We therefore consider the often-used term ECG Imaging (ECGI) unfortunate. It suggests a similarity to other medical imaging modalities, where the quality of the image represents the accuracy of the data. For instance, echocardiograms and PET scan are much less crisp than CT images, corresponding to the lower accuracy of these methods. ECGI “images” are crisp, high-resolution plots of isopotential lines or isochrones on the heart, falsely suggesting a high accuracy of the data. We have been pondering on how to visualize the uncertainty in images that represent the results of inverse electrocardiography, but so far, we failed to come up with a solution. Realistic expectations of the accomplishments of inverse electrocardiography would be served by avoiding the term ECGI and taking the shortcomings and strengths of each of the methods, and how these influence accuracy, into account.

Researchers working on the inverse problem of the ECG often get the question: “why do not you simply use machine learning”? There are many applications of machine learning on the ECG, but they mainly concern ECG classification (Trayanova et al., 2021). Machine learning in general has made strong progress in the recent years in many fields. So far, there are just a few studies on the use of machine learning for inverse ECG (Bacoyannis et al., 2021; Chen et al., 2022). One disadvantage, in our view, is that it is a black box: it is not clear what has actually been learned. What an AI-trained algorithm will do on ECG patterns it has not been trained for is unclear. The clinical performance of deep learning in inverse ECG mapping remains to be established. However, we can imagine benefit in combining deep learning with the two source models discussed in this review, for instance in the choice of the regularization parameters or the relative weights in the two approaches in a combined EP-EDL inverse.

In summary, both the EP- and EDL-based method have advantages and disadvantages. The main advantage of the EDL-based inverse is that it also provides activation and repolarization times at the endocardium. There are clinically relevant abnormalities for which the EDL inverse cannot readily be used, such as acute ischemia and atrial/ventricular fibrillation, which would require the use of the EP-based method. We make some suggestions on how the EDL- and EP-based methods can combine forces and reduce error. Thus, the two inverse methods are at least in part complementary. This feeds the expectation that combination of the two methods yields better results than each method does separately.

## References

[B1] Bacoyannis T. Ly B. Cedilnik N. Cochet H. Sermesant M. (2021). Deep learning formulation of electrocardiographic imaging integrating image and signal information with data-driven regularization. Europace 23, I55–I62. 10.1093/europace/euaa391 33751073

[B2] Bakker J. M. de Van Capelle F. J. Janse M. J. Wilde A. A. Coronel R. Becker A. E. (1988). Reentry as a cause of ventricular tachycardia in patients with chronic ischemic heart disease: electrophysiologic and anatomic correlation. Circulation 77, 589–606. 10.1161/01.cir.77.3.589 3342490

[B3] Barr R. C. Ramsey M. Spach M. S. (1977). Relating epicardial to body surface potential distributions by means of transfer coefficients based on geometry measurements. IEEE Trans. Biomed. Eng. 24, 1–11. 10.1109/TBME.1977.326201 832882

[B4] Bear L. R. Bouhamama O. Cluitmans M. Duchateau J. Walton R. D. Abell E. (2019a). Advantages and pitfalls of noninvasive electrocardiographic imaging. J. Electrocardiol. 57, S15–S20. 10.1016/j.jelectrocard.2019.08.007 31477238

[B5] Bear L. R. Cluitmans M. Abell E. Rogier J. Labrousse L. Cheng L. K. (2021). Electrocardiographic imaging of repolarization abnormalities. J. Am. Heart Assoc. 10, e020153. 10.1161/JAHA.120.020153 33880931 PMC8200734

[B6] Bear L. R. Huntjens P. R. Walton R. D. Bernus O. Coronel R. Dubois R. (2018b). Cardiac electrical dyssynchrony is accurately detected by noninvasive electrocardiographic imaging. Heart rhythm. 15, 1058–1069. 10.1016/j.hrthm.2018.02.024 29477975

[B7] Bear L. R. LeGrice I. J. Sands G. B. Lever N. A. Loiselle D. S. Paterson D. J. (2018a). How accurate is inverse electrocardiographic mapping? Circ. Arrhythm. Electrophysiol. 11, e006108. 10.1161/CIRCEP.117.006108 29700057

[B8] Bear L. R. Walton R. D. Abell E. Coudière Y. Haissaguerre M. Bernus O. (2019b). Optical imaging of ventricular action potentials in a torso tank: a new platform for non-invasive electrocardiographic imaging validation. Front. Physiol. 10, 146. 10.3389/fphys.2019.00146 30863318 PMC6399141

[B9] Boonstra M. J. Roudijk R. W. Brummel R. Kassenberg W. Blom L. J. Oostendorp T. F. (2022). Modeling the his-purkinje effect in non-invasive estimation of endocardial and epicardial ventricular activation. Ann. Biomed. Eng. 50, 343–359. 10.1007/s10439-022-02905-4 35072885 PMC8847268

[B10] Borràs M. Chamorro-Servent J. (2021). Electrocardiographic imaging: a comparison of iterative solvers. Front. Physiol. 12, 620250. 10.3389/fphys.2021.620250 33613311 PMC7886787

[B11] Chamorro-Servent J. Dubois R. Coudière Y. (2019). Considering new regularization parameter-choice techniques for the Tikhonov method to improve the accuracy of electrocardiographic imaging. Front. Physiol. 10, 273. 10.3389/fphys.2019.00273 30971937 PMC6445955

[B12] Chen K.-W. Bear L. Lin C.-W. (2022). Solving inverse electrocardiographic mapping using machine learning and deep learning frameworks. Sensors 22, 2331. 10.3390/s22062331 35336502 PMC8951148

[B13] Cheng L. K. Bodley J. M. Pullan A. J. (2003). Comparison of potential- and activation-based formulations for the inverse problem of electrocardiology. IEEE Trans. Biomed. Eng. 50, 11–22. 10.1109/TBME.2002.807326 12617520

[B14] Child N. Bishop M. J. Hanson B. Coronel R. Opthof T. Boukens B. J. (2015). An activation-repolarization time metric to predict localized regions of high susceptibility to reentry. Heart rhythm. 12, 1644–1653. 10.1016/j.hrthm.2015.04.013 25863160 PMC4717521

[B15] Cluitmans M. Brooks D. H. MacLeod R. Dössel O. Guillem M. S. Van Dam P. M. (2018). Validation and opportunities of electrocardiographic imaging: from technical achievements to clinical applications. Front. Physiol. 9, 1305. 10.3389/fphys.2018.01305 30294281 PMC6158556

[B16] Cluitmans M. Coll-Font J. Erem B. Bear L. Nguyên U. C. Ter Bekke R. T. (2022). Spatiotemporal approximation of cardiac activation and recovery isochrones. J. Electrocardiol. 71, 1–9. 10.1016/j.jelectrocard.2021.12.007 34979408

[B17] Cluitmans M. Volders P. (2017). Influence of body-surface geometry accuracy on noninvasive reconstruction of electrical activation and recovery in electrocardiographic imaging. Comput. Cardiol., 1–4. 10.22489/CinC.2017.243-189

[B18] Cluitmans M. J. M. Bonizzi P. Karel J. M. H. Das M. Kietselaer BLJH Jong Mmj de (2017a). In Vivo validation of Electrocardiographic Imaging. JACC Clin. Electrophysiol. 3, 232–242. 10.1016/j.jacep.2016.11.012 29759517

[B19] Cluitmans M. J. M. Clerx M. Vandersickel N. Peeters R. L. M. Volders P. G. A. Westra R. L. (2017b). Physiology-based regularization of the electrocardiographic inverse problem. Med. Biol. Eng. Comput. 55, 1353–1365. 10.1007/s11517-016-1595-5 27873155 PMC5544815

[B20] Colli-Franzone P. Guerri L. Tentoni S. Viganotti C. Baruffi S. Spaggiari S. (1985). A mathematical procedure for solving the inverse potential problem of electrocardiography. analysis of the time-space accuracy from *in vitro* experimental data. Math. Biosci. 77, 353–396. 10.1016/0025-5564(85)90106-3

[B21] Coronel R. de Bakker J. M. T. Wilms-Schopman F. J. G. Opthof T. Linnenbank A. C. Belterman C. N. (2006). Monophasic action potentials and activation recovery intervals as measures of ventricular action potential duration: experimental evidence to resolve some controversies. Heart rhythm. 3, 1043–1050. 10.1016/j.hrthm.2006.05.027 16945799

[B22] Coumel P. (1987). The management of clinical arrhythmias. An overview on invasive versus non-invasive electrophysiology. Eur. Heart J. 8, 92–99. 10.1093/oxfordjournals.eurheartj.a062259 2436917

[B23] Cuculich P. S. Zhang J. Wang Y. Desouza K. A. Vijayakumar R. Woodard P. K. (2011). The electrophysiological cardiac ventricular substrate in patients after myocardial infarction: noninvasive characterization with electrocardiographic imaging. J. Am. Coll. Cardiol. 58, 1893–1902. 10.1016/j.jacc.2011.07.029 22018301 PMC3365586

[B24] Cuppen J. J. M. Van Oosterom A. (1984). Model studies with the inversely calculated isochrones of ventricular depolarization. IEEE Trans. Biomed. Eng. 31, 652–659. 10.1109/TBME.1984.325315 6490025

[B25] Duchateau J. Potse M. Dubois R. (2017). Spatially coherent activation maps for electrocardiographic imaging. IEEE Trans. Biomed. Eng. 64, 1149–1156. 10.1109/TBME.2016.2593003 27448338

[B26] Duchateau J. Sacher F. Pambrun T. Derval N. Chamorro-Servent J. Denis A. (2019). Performance and limitations of noninvasive cardiac activation mapping. Heart rhythm. 16, 435–442. 10.1016/j.hrthm.2018.10.010 30385382

[B27] Eichenlaub M. Mueller-Edenborn B. Lehrmann H. Minners J. Nairn D. Loewe A. (2021). Non-invasive body surface electrocardiographic imaging for diagnosis of atrial cardiomyopathy. Europace 23, 2010–2019. 10.1093/europace/euab140 34463710

[B28] Gardner P. I. Ursell P. C. Fenoglio J. J. Wit A. L. (1985). Electrophysiologic and anatomic basis for fractionated electrograms recorded from healed myocardial infarcts. Circulation 72, 596–611. 10.1161/01.cir.72.3.596 4017211

[B29] Geselowitz D. B. (1992). Description of cardiac sources in anisotropic cardiac muscle. Application of bidomain model. J. Electrocardiol. 25, 65–67. 10.1016/0022-0736(92)90063-6 1297711

[B30] Ghanem R. N. Jia P. Ramanathan C. Ryu K. Markowitz A. Rudy Y. (2005). Noninvasive electrocardiographic imaging (ECGI): comparison to intraoperative mapping in patients. Heart rhythm. 2, 339–354. 10.1016/j.hrthm.2004.12.022 15851333 PMC1949041

[B31] Graham A. J. Orini M. Zacur E. Dhillon G. Daw H. Srinivasan N. T. (2019). Simultaneous comparison of electrocardiographic imaging and epicardial contact mapping in structural heart disease. Circ. Arrhythm. Electrophysiol. 12, e007120. 10.1161/CIRCEP.118.007120 30947511

[B32] Gulrajani R. M. (1998). The forward and inverse problems of electrocardiography. IEEE Eng. Med. Biol. Mag. 17, 84–122. 10.1109/51.715491 9770610

[B33] Haïssaguerre M. Hocini M. Cheniti G. Duchateau J. Sacher F. Puyo S. (2018). Localized structural alterations underlying a subset of unexplained sudden cardiac death. Circ. Arrhythm. Electrophysiol. 11, 0061200–e6212. 10.1161/CIRCEP.117.006120 PMC7661047 30002064

[B34] Hansen P. C. O’Leary D. P. (1993). The use of the L-curve in the regularization of discrete ill-posed problems. SIAM J. Sci. Comput. 14, 1487–1503. 10.1137/0914086

[B35] Haws C. W. Lux R. L. (1990). Correlation between *in vivo* transmembrane action potential durations and activation-recovery intervals from electrograms. Effects of interventions that alter repolarization time. Circulation 81, 281–288. 10.1161/01.cir.81.1.281 2297832

[B36] Hohmann S. Rettmann M. E. Konishi H. Borenstein A. Wang S. Suzuki A. (2019). Spatial accuracy of a clinically established noninvasive electrocardiographic imaging system for the detection of focal activation in an intact porcine model. Circ. Arrhythm. Electrophysiol. 12, e007570. 10.1161/CIRCEP.119.007570 31707808

[B37] Huiskamp G. Van Oosterom A. (1988). The depolarization sequence of the human heart surface computed from measured body surface potentials. IEEE Trans. Biomed. Eng. 35, 1047–1058. 10.1109/10.8689 3220498

[B38] Janse M. J. Wit A. L. (1989). Electrophysiological mechanisms of ventricular arrhythmias resulting from myocardial ischemia and infarction. Physiol. Rev. 69, 1049–1169. 10.1152/physrev.1989.69.4.1049 2678165

[B39] Janssen A. M. Potyagaylo D. Dössel O. Oostendorp T. F. (2018). Assessment of the equivalent dipole layer source model in the reconstruction of cardiac activation times on the basis of BSPMs produced by an anisotropic model of the heart. Med. Biol. Eng. Comput. 56, 1013–1025. 10.1007/s11517-017-1715-x 29130137 PMC5978848

[B40] Jelvehgaran P. O’Hara R. Prakosa A. Chrispin J. Boink G. J. J. Trayanova N. (2023). Computational Re-entry vulnerability index mapping to guide ablation in patients with postmyocardial infarction ventricular tachycardia. JACC Clin. Electrophysiol. 9, 301–310. 10.1016/j.jacep.2022.10.002 36752477

[B41] Jiang M. Xia L. Shou G. Wei Q. Liu F. Crozier S. (2009). Effect of cardiac motion on solution of the electrocardiography inverse problem. IEEE Trans. Biomed. Eng. 56, 923–931. 10.1109/TBME.2008.2005967 19272916

[B42] Johnston P. R. Gulrajani R. M. (1997). A new method for regularization parameter determination in the inverse problem of electrocardiography. IEEE Trans. Biomed. Eng. 44, 19–39. 10.1109/10.553710 9214781

[B43] MacLeod R. S. Ni Q. Punske B. Ershler P. R. Yilmaz B. Taccardi B. (2000). Effects of heart position on the body-surface electrocardiogram. J. Electrocardiol. 33, 229–237. 10.1054/jelc.2000.20357 11265726

[B44] Marquardt D. W. (1963). An algorithm for least-squares estimation of nonlinear parameters. J. Soc. Industrial Appl. Math. 11, 431–441. 10.1137/0111030

[B45] Martin R. O. Pilkington T. C. (1972). Unconstrained inverse electrocardiography: epicardial potentials. IEEE Trans. Biomed. Eng. 19, 276–285. 10.1109/TBME.1972.324070 5036139

[B46] Milanič M. Jazbinšek V. MacLeod R. S. Brooks D. H. Hren R. (2014). Assessment of regularization techniques for electrocardiographic imaging. J. Electrocardiol. 47, 20–28. 10.1016/j.jelectrocard.2013.10.004 24369741 PMC4154607

[B47] Nabauer M. Beuckelmann D. J. Uberfuhr P. Steinbeck G. (1996). Regional differences in current density and rate-dependent properties of the transient outward current in subepicardial and subendocardial myocytes of human left ventricle. Circulation 93, 168–177. 10.1161/01.cir.93.1.168 8616924

[B48] Oostendorp T. Nenonen J. Korhonen P. (2002). Noninvasive determination of the activation sequence of the heart: application to patients with previous myocardial infarctions. J. Electrocardiol. 35, 75–80. 10.1054/jelc.2002.37158 12539102

[B49] Oostendorp T. F. Van Oosterom A. Huiskamp G. (1989). Interpolation on a triangulated 3D surface. J. Comput. Phys. 80, 331–343. 10.1016/0021-9991(89)90103-4

[B50] Oosterhoff P. Meijborg V. M. F. Dam van P. M. Dessel van PFHM Belterman C. N. W. Streekstra G. J. (2016). Experimental validation of noninvasive epicardial and endocardial activation imaging. Circ. Arrhythm. Electrophysiol. 9, e004104. 10.1161/CIRCEP.116.004104 27439651

[B51] Opthof T. Janse M. J. Meijborg V. M. F. Cinca J. Rosen M. R. Coronel R. (2016). Dispersion in ventricular repolarization in the human, canine and porcine heart. Prog. Biophys. Mol. Biol. 120, 222–235. 10.1016/j.pbiomolbio.2016.01.007 26790342

[B52] Orini M. Graham A. J. Srinivasan N. T. Campos F. O. Hanson B. M. Chow A. (2020). Evaluation of the reentry vulnerability index to predict ventricular tachycardia circuits using high-density contact mapping. Heart rhythm. 17, 576–583. 10.1016/j.hrthm.2019.11.013 31751771 PMC7105818

[B53] Pereira H. Niederer S. Rinaldi C. A. (2020). Electrocardiographic imaging for cardiac arrhythmias and resynchronization therapy. Europace 22, 1447–1462. 10.1093/europace/euaa165 32754737 PMC7544539

[B54] Plonsey R. Barr R. C. (1987). Mathematical modeling of electrical activity of the heart. J. Electrocardiol. 20, 219–226. 10.1016/s0022-0736(87)80019-5 3309111

[B55] Potse M. Vinet A. Opthof T. Coronel R. (2009). Validation of a simple model for the morphology of the T wave in unipolar electrograms. Am. J. Physiol. Heart Circ. Physiol. 297, H792–H801. 10.1152/ajpheart.00064.2009 19465555

[B56] Roudijk R. W. Boonstra M. J. Brummel R. Kassenberg W. Blom L. J. Oostendorp T. F. (2021). Comparing non-invasive inverse electrocardiography with invasive endocardial and epicardial electroanatomical mapping during sinus rhythm. Front. Physiol. 12, 730736. 10.3389/fphys.2021.730736 34671274 PMC8521153

[B57] Rudy Y. (1999). Electrocardiographic imaging: a noninvasive imaging modality for characterization of intramural myocardial activation. J. Electrocardiol. 32, 1–6. 10.1016/s0022-0736(99)90025-0 10688295

[B58] Santangeli P. Marchlinski F. E. (2016). Substrate mapping for unstable ventricular tachycardia. Heart rhythm. 13, 569–583. 10.1016/j.hrthm.2015.09.023 26410105

[B59] Sapp J. L. Dawoud F. Clements J. C. Horáček B. M. (2012). Inverse solution mapping of epicardial potentials: quantitative comparison with epicardial contact mapping. Circ. Arrhythm. Electrophysiol. 5, 1001–1009. 10.1161/CIRCEP.111.970160 22923272

[B60] Schuler S. Schaufelberger M. Bear L. R. Bergquist J. A. Cluitmans M. J. M. Coll-Font J. (2022). Reducing line-of-block artifacts in cardiac activation maps estimated using ECG imaging: a comparison of source models and estimation methods. IEEE Trans. Biomed. Eng. 69, 2041–2052. 10.1109/TBME.2021.3135154 34905487

[B61] Shivkumar K. (2019). Catheter ablation of ventricular arrhythmias. N. Engl. J. Med. 380, 1555–1564. 10.1056/NEJMra1615244 30995375

[B62] Stevenson W. G. Delacretaz E. Friedman P. L. Ellision K. E. (1998). Identification and ablation of macroreentrant ventricular tachycardia with the CARTO electroanatomical mapping system. Pacing Clin. Electrophysiol. 21, 1448–1456. 10.1111/j.1540-8159.1998.tb00217.x 9670190

[B63] Stevenson W. G. Khan H. Sager P. Saxon L. A. Middlekauff H. R. Natterson P. D. (1993). Identification of reentry circuit sites during catheter mapping and radiofrequency ablation of ventricular tachycardia late after myocardial infarction. Circulation 88, 1647–1670. 10.1161/01.cir.88.4.1647 8403311

[B64] Stevenson W. G. Soejima K. (2007). Catheter ablation for ventricular tachycardia. Circulation 115, 2750–2760. 10.1161/CIRCULATIONAHA.106.655720 17533195

[B65] Tate J. D. Good W. W. Zemzemi N. Boonstra M. Van Dam P. Brooks D. H. Uncertainty quantification of the effects of segmentation variability in ECGI. 2021. p. 515–522. 10.1007/978-3-030-78710-3_49 PMC9019843 35449797

[B66] Trayanova N. A. Popescu D. M. Shade J. K. (2021). Machine learning in arrhythmia and electrophysiology. Circ. Res. 128, 544–566. 10.1161/CIRCRESAHA.120.317872 33600229 PMC7899082

[B67] Umapathy K. Nair K. Masse S. Krishnan S. Rogers J. Nash M. P. (2010). Phase mapping of cardiac fibrillation. Circ. Arrhythm. Electrophysiol. 3, 105–114. 10.1161/CIRCEP.110.853804 20160178

[B68] Van Dam P. M. Oostendorp T. F. Linnenbank A. C. Van Oosterom A. (2009). Non-invasive imaging of cardiac activation and recovery. Ann. Biomed. Eng. 37, 1739–1756. 10.1007/s10439-009-9747-5 19562487 PMC2721141

[B69] Van der Waal J. Meijborg V. Boonstra M. Oostendorp T. Coronel R. (2022). “On the initial estimate of repolarization times for inverse reconstruction using the equivalent dipole layer source model,” in 2022 Computing in Cardiology Conference (CinC).

[B70] Van der Waal J. G. Meijborg V. M. F. Belterman C. N. W. Streekstra G. J. Oostendorp T. F. Coronel R. (2021). *Ex vivo* validation of noninvasive epicardial and endocardial repolarization mapping. Front. Physiol. 12, 1–11. 10.3389/fphys.2021.737609 PMC8569864 34744778

[B71] Van Oosterom A. (2004). The dominant T wave. J. Electrocardiol. 37, 193–197. 10.1016/j.jelectrocard.2004.08.056 15534840

[B72] Van Oosterom A. (2014). A comparison of electrocardiographic imaging based on two source types. Europace 16, iv120–8. 10.1093/europace/euu268 25362162

[B73] Van Oosterom A. Huiskamp G. J. (1989). The effect of torso inhomogeneities on body surface potentials quantified using “tailored” geometry. J. Electrocardiol. 22, 53–72. 10.1016/0022-0736(89)90023-x 2921579

[B74] Wang L. Gharbia O. A. Nazarian S. Horáček B. M. Sapp J. L. (2018). Non-invasive epicardial and endocardial electrocardiographic imaging for scar-related ventricular tachycardia. EP Eur. 20, f263–f272. 10.1093/europace/euy082 PMC6140441 29684187

