# Supplementary material for: Basis and applicability of noninvasive inverse electrocardiography: a comparison between cardiac source models
Source: Front Physiol. 2023 Dec 13;14:1295103. doi: 10.3389/fphys.2023.1295103 (PMC10752226; doi:10.3389/fphys.2023.1295103)
Supplement: Supplementary file 1 [file DataSheet1.PDF]

Supplementary Material

S.1. Details on the constructions of the transfer matrices

S.1.1 Epicardial Surface Model

In addition to Equation 1 of the paper of which this is the supplementary material, which reflects the electrical propagation in the volume of the torso, three conditions apply to the boundaries. The two first conditions apply on the torso surface: a first equation provides the value *V* of the electrical potentials at body surface (Dirichlet conditions). The second equation, known as the zero normal flux condition, reflects that the air outside the torso surface is assumed to have a zero conductivity and thus no charge can leave it:

$$\begin{cases} \varphi(x_T)) = V,\ x_T \text{ on the torso boundary} \\ \frac{d\varphi(x_T)}{dn} = 0,\ \mathrm{x_T} \text{ on the torso boundary} \end{cases} \tag{S.1}$$

with *n* the normal vector to the body surface. The third condition applies with the heart boundary. The mathematical formulation of the latter depends on the chosen cardiac source model.

Since there is no active electrical source present between the epicardium to the surface of the body, Ohm's law applies in the torso volume. This can be written in its local form at any time *t* under the quasi-static assumption:

$$\nabla \cdot \left(\sigma \nabla \varphi(x)\right) = 0 \tag{S.2}$$

With $\varphi(x)$ the potential in any point x within the torso domain and σ the conductivity of the torso which is a tensor in its most generic form. Equation 1 is independent of the equivalent source model used for the heart.

Let $\varphi(x_H)$ be the potential at any point x_H of that boundary, Equation S.3 is the third condition that applies to the potential φ with the heart boundary:

$$\varphi(x_H) = \varphi_H, x_H \text{ on the heart boundary} \tag{S.3}$$

Equation 2 of the paper together with the three boundary conditions lead to the following system of equations for the EP formulation:

$$\begin{cases} \nabla \cdot \left(\sigma \nabla \varphi(x)\right) = 0 \\ \varphi(x_T)) = V, x_T \text{ on the torso boundary} \\ \dfrac{d\varphi(x_T)}{dn} = 0, x_T \text{ on the torso boundary} \\ \varphi(x_H) = \varphi_H, x_H \text{ on the heart boundary} \end{cases} \tag{S.4}$$

Numerical methods are used to solve the above equations. Using a volume conductor model, discretization can be obtained by meshing the torso domain using either the boundary element method (BEM)[1,2], the finite element method (FEM) or an efficient meshless method[3]. This results in the equation that relates V_i, the potential at electrode i at body surface, to the potentials at all N_h nodes of the myocardial surface mesh φ_j:

$$V_i(t) = \sum_{j=1}^{N_h} T_{ij}\, \varphi_j(t) \tag{S.5}$$

In this equation, T_{ij} is the potential at electrode i if at the epicardium node j has potential 1, and all other nodes have potential 0. This is also equation 2 of the paper of which this is the supplementary material.

S.1.2 Equivalent Dipole Layer Model

In the EDL model, there are no boundary conditions at the heart surface, so S.1 are the only boundary conditions. Whereas there are no electric source in the EP model, there are sources in the EDL model: a dipole layer at the epicardial surface, with a strength that is proportional to the local transmembrane potential and a direction that is perpendicular to the surface[4]. As for the EP model, there are numerical methods to solve the potential generated by these sources in a volume conductor model[5]. This results in the equation that relates the equivalent dipole layer strength $d_j(t)$ at the myocardial surface to the potentials at the surface electrodes:

$$V_i(t) = \sum_{j=1}^{N_h} A_{ij}\, d_j(t) \tag{S.5}$$

In this equation, A_{ij} is the potential at electrode i if myocardial surface node j is completely depolarized, while all other nodes are completely depolarized. This is also Equation 4 of the paper of which this is the supplementary material.

S2. Estimation of transmembrane potential waveform in EDL model

Van Oosterom introduced the concept of the dominant T-wave to reconstruct the wave form of the transmembrane potential (TMP) from the shape of the T-wave in the ECG at the body surface[6]. The dominant T-wave represents the overall waveform of the T-wave in a body surface potential map. Figure 1A depicts how the dominant T-wave is constructed. First, the Root-Mean-Square (RMS) average of the ECG over all leads is determined (dotted red line in figure 1A). The central part of the dominant T-wave is defined as the RMS of the ECG from the J-point to the moment of fastest downward slope (dashed red). The start of the dominant T-wave is a spline from zero potential at the moment of QRS-onset to the start of the central part (dashed blue). The end is a spline from the end of the central part to zero potential at the end of the T-wave (dashed blue).

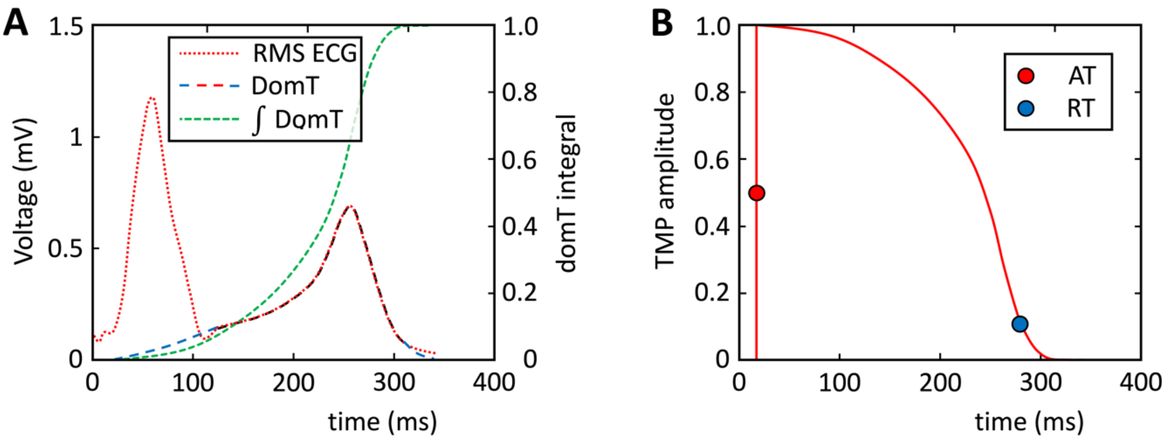

Figure 1: Construction of the template for the repolarization waveform from the observed T-wave. A: Root-Mean-Square (RMS) of a body surface ECG (dotted red), the dominant T-wave (dashed red/blue) and integral of the

dominant T-wave, scaled to 1 at the maximum (dashed green). B: The TMP waveform template (1 minus the integral of the dominant T-wave). The activation time (AT) of the moment of upstroke, the repolarization time (RT) is defined as the moment at which the TMP is down to 10% of its maximum.

Van Oosterom argued that the repolarization waveform of the transmembrane potential follows the negative of the integral of the dominant T-wave[6,7]. Figure 1A shows the integral of the dominant T-wave, scaled to a maximum of 1 (arbitrary units). Figure 1B shows the template for the TMP waveform, scaled from zero in resting state to 1 at the moment of depolarization, and subsequently following 1 minus the integral of the dominant T-wave. The repolarization time is defined the moment at which the TMP is down to 10% of its maximum.

In the EDL model, this template is shifted and stretched for each source node j at the myocardial surface to match the activation time τ_j and repolarization time ρ_j of that node, resulting in a dipole layer waveform per node that depends on the timing of that node: $d(\tau_j, \rho_j, t)$ (see Figure 3 of the main paper).

Supplementary References

1. Barr RC, Ramsey M, Spach MS. Relating Epicardial to Body Surface Potential Distributions by Means of Transfer Coefficients Based on Geometry Measurements. *IEEE Trans Biomed Eng* 1977;**24**:1–11.
2. Gulrajani RM. The forward and inverse problems of electrocardiography. *IEEE Engineering in Medicine and Biology Magazine* 1998;**17**:84–101, 122.
3. Wang Y, Rudy Y. Application of the Method of Fundamental Solutions to Potential-based Inverse Electrocardiography. *Ann Biomed Eng* 2006;**34**:1272–88.
4. Geselowitz DB. Description of cardiac sources in anisotropic cardiac muscle. *J Electrocardiol* 1992;**25**:65–7.
5. Huiskamp G, Oosterom A van. The depolarization sequence of the human heart surface computed from measured body surface potentials. *IEEE Trans Biomed Eng* 1988;**35**:1047–58.
6. Oosterom A van. The dominant T wave. *J Electrocardiol* 2004;**37**:193–7.
7. Dam PM Van, Oostendorp TF, Linnenbank AC, Oosterom A Van. Non-invasive imaging of cardiac activation and recovery. *Ann Biomed Eng* 2009;**37**:1739–56.
